# Challenge of Incorporating New Drugs for Breast Cancer in Brazil: A Proposed Framework for Improving Access to Innovative Therapies

**DOI:** 10.1200/GO.20.00566

**Published:** 2021-04-06

**Authors:** Carlos Barrios, Ruffo Freitas-Junior, Sandro Martins, Jose Bines, Maria Del Pilar Estevez-Diz, Maira Caleffi

**Affiliations:** ^1^ Grupo Oncoclínicas, Latin American Cooperative Oncology Group (LACOG), Porto Alegre, Brazil; ^2^ CORA, Advanced Center for Diagnosis of Breast Diseases, Federal University of Goias, Goiânia, Brazil; ^3^ Araujo Jorge Hospital of Associação de Combate ao Câncer em Goiás, Goiânia, Brazil; ^4^ Medical Oncology Unit, University Hospital of Brasília/EBSERH, Brasília, Brazil; ^5^ Clínica São Vicente, Rio de Janeiro, Brazil; ^6^ Instituto do Câncer do Estado de São Paulo/Faculdade de Medicina da Universidade de São Paulo, Onco Star Rede D'Or, São Paulo, Brazil; ^7^ Hospital Moinhos de Vento, Femama, Porto Alegre, Brazil

## Abstract

**PURPOSE:**

The objective of this review is to address the barriers limiting access to treatment of advanced metastatic breast cancer (mBC) in Brazil, specifically for patients in the public health care system, arguably those with the least access to innovation.

**MATERIALS AND METHODS:**

A selected panel of Brazilian experts in BC were provided with a series of relevant questions to address in a multiday conference. During the conference, responses were discussed and edited by the entire group through numerous drafts and rounds of discussion until a consensus was achieved.

**RESULTS:**

The authors propose specific and realistic recommendations for implementing access to new drugs in cancer care in Brazil. Moreover, in creating these recommendations and framework, the authors strive to address the most important barriers and impediments for technology incorporation. A feasible and specific multidisciplinary process is proposed, which is based on the collective participation of all involved stakeholders.

**CONCLUSION:**

Given the current benefits and likely future developments, there is a great need to expand treatments for mBC not only in Brazil but also in most other countries in the world where access issues remain an unresolved demand. Adapting the current framework is essential for accomplishing this goal. The recommendations in this review can serve as a framework for adoption of new technologies in countries with limited resources.

## INTRODUCTION

Breast cancer (BC) represents a substantial health care problem worldwide and a major burden for Latin America where incidence rates have increased at a greater rate than in developed nations over the last few decades.^1,2^ In Brazil, it is the most frequent cancer in women, with 66,280 new cases per year and an incidence rate of 61.6 per 100,000 women, numbers that are expected to double by 2035.^3,4^ Approximately 17,000 women die from BC each year, translating to a mortality rate of 16.2 per 100,000 women.^3^ Although incidence rates are lower than in more developed countries, mortality rates are similar (16.2 in Brazil *v* 14.9 in the United States and 12.5 in Norway).^3,5^ Mortality rates have remained stable in the last few decades.^3,6^ Although clinical trials have demonstrated significantly better outcomes with the introduction of new drugs for the treatment of BC in recent years, particularly with approaches directed to specific populations, these results are yet to be translated to better results in the general population. Within this context, we will concentrate in addressing the challenge of incorporating new drugs for advanced BC, addressing the Brazilian regulatory scenario, and proposing a framework with potential applicability to similar developing societies.

**CONTEXT**

**Key Objective**
While recognizing that there is no simple or better solution to this problem, we propose a framework that involves all potential stakeholders in the process to address the incorporation of innovative therapies.
**Knowledge Generated**
Access to new technologies and medicines is a universal challenge for health care systems worldwide and one of the major reasons for discrepancies that compromise cancer care outcomes. We exemplify the situation, addressing the incorporation of new drugs for the management of patients with hormone receptor–positive breast cancer in Brazil.
**Relevance**
Although any potential solution to this complex issue should be tempered by the particular situation in each individual country, this framework should be seen as applicable to other low-resource scenarios. Ultimately, to be successful, we need to include all interested parties in a clear, very transparent, and predefined process, all aligned with the final objective of achieving the best possible outcome for our patients.

## MATERIALS AND METHODS

The Americas Health Foundation (AHF) identified clinicians and scientists with an academic or hospital affiliation who are experts in the field and who have published in the hormone receptor–positive (HR+) and human epidermal growth factor receptor 2–negative (HER2−) metastatic BC (mBC) arena since 2014. As a result, AHF convened a six-member panel of clinical and scientific experts from Brazil. Great attention was paid to ensure a diverse group representing various disciplines related to HR+ and HER2− mBC. To better focus the discussion, AHF staff independently developed specific questions, addressing the salient issues on the subject, for the panel to address. A written response to each question was initially drafted by a different member of the panel. During the multiday meeting of the panel, each narrative was discussed and edited by the entire group, through numerous drafts and rounds of discussion until complete consensus was obtained. The objective of this article is to create a practical document addressing the framework for adoption of new technologies for patients with HR+ and HER2− mBC in Brazil.

### Search Strategy and Selection Criteria

Manuscripts referenced in this consensus paper were identified through searches of PubMed and Embase with the search terms metastatic breast cancer, breast cancer in Brazil, and HR+ and HER2− from November 2014 to November 2019. Articles were also identified through the bibliographies of the papers identified in the search and from sources of the authors' own files. Particular attention was paid to papers that reviewed or summarized the topic in question or that were related to activities in the public health system of Brazil. The final reference list was generated on the basis of the relevance to the broad scope of this consensus document.

### Health Systems and BC Care in Brazil

In Brazil, although approximately one quarter of the population can afford private health care, or Supplementary Health (SH), 77% of Brazilians depend exclusively on the public health system.^7^ The latter, “Sistema Único de Saúde” (SUS), has been responsible for important advancements in health coverage with significant impact in some areas such as communicable diseases.^8^ However, important differences are identified between the two systems, reflecting inequality and directly affecting cancer care and patient outcomes.^9^

The national policy for cancer control in Brazil was proposed just in the last decade and establishes comprehensive actions for the continuum of cancer management, organizing regional networks to integrate all levels of care.^10^ Some of the challenges that these networks face include (1) fragmentation of care, (2) lack of awareness of specific needs of this patient population, and (3) overwhelming responsibilities that overburden primary clinics, which are not evenly distributed throughout the country.

As in most developing countries, health care in Brazil is unevenly distributed. The regions with the most qualified health care facilities are in the South and the Southeastern Brazil, accounting for more than two thirds of centers, as compared with three other regions that have < 10% each.^11^ This inequality in resource distribution leads to important restrictions in diagnosis, staging, and comprehensive treatment. Additionally, conflicting regulations in specific areas of cancer care, insufficient funding of cancer programs, weak epidemiologic surveillance, and unclear pathways for patients with cancer increase barriers for adequate care.

Furthermore, because of historical and complex racial composition, the prevalence of BC subtypes differs among the country's regions.^12,13^ The North and Northeast regions have a higher proportion of triple-negative BC, whereas the South and Southeast have a greater prevalence of luminal A and HER2+ subtypes. Additionally, although in the SH system, 80% of BC are diagnosed in stages I-II, only 60% of cases in the public system have early-stage disease.^14^ Importantly as well, different patterns of BC care for women with or without health plans coexist locally. Considering all these discrepancies, it is not surprising that in Brazil, premature death and disability because of BC are higher than those reported in developed countries.

Furthermore, the disease has a substantial economic impact, both directly from treatment-related costs and indirectly because of loss of productivity in the workplace. Total costs of BC generally increase with the advancement of the disease stage at diagnosis.^15^ Comprehensive data on the costs associated with advanced BC in Brazil are lacking. Additionally, costs are compounded by an increase in judicialization, a process where patients request coverage for expensive, regularly not available therapies in SUS or even the SH, through the judiciary system.^16^

All these discrepancies result in lower-quality care for women, particularly those in a state of vulnerability, with lower availability and access to health care professionals and services. Negligent and discriminatory services and prioritization of private insurance over public health system users are added problems that expand discrepancies and affect outcomes.^17,18^ Additionally, approximately 38.1 million Brazilians live in poverty, the majority of which belong to the Black or brown population.^12^ Clearly, these ethno-racial and social disparities in early detection, presentation, and management of BC must be considered within a broader perspective, and a number of initiatives have been proposed to reduce these gaps.^19,20^

Health equity is shaped by the distribution of the social determinants of health.^21^ Regardless of income classification, wide disparities in health status exist across social groups depending on their socioeconomic status (SES). There is ample evidence that SES, including education, employment status, income level, sex, and ethnicity, determines the health of an individual. In all countries, regardless of income classification, wide disparities can be identified across the health status of social groups based on SES. The lower the SES, the higher their risk of experiencing poor health outcomes.^22^ There is an abundance of economic indexes measuring inequalities among different countries and populations.^23-25^ Indexes concerning SES inequalities in BC care in Brazil are shown in Table 1. Importantly as well, available data call attention to the negative impact of metastatic disease on patient quality of life, especially in the vulnerable population, because of long commutes, lack of basic comforts throughout treatment, and increased risk of unemployment.^17,60^

**TABLE 1:**
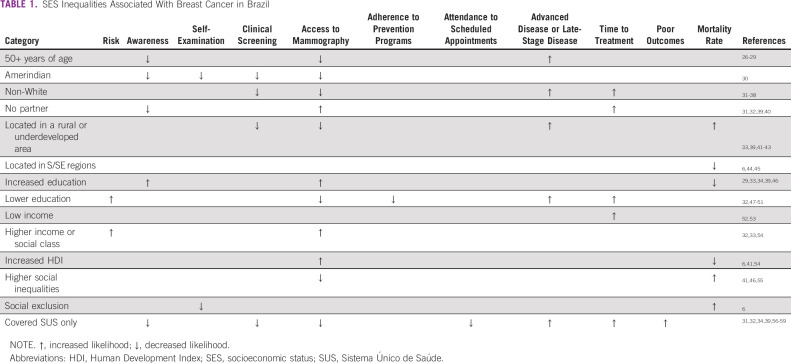
SES Inequalities Associated With Breast Cancer in Brazil

### HR+ and HER2− mBC in Brazil

HR+ and HER2− mBC is the most frequent form of the disease. Median overall survival (OS) has been documented at 30-45 months and has remained stable over the last few decades.^9^ In Brazil, precise information regarding real numbers, specific characteristics, and the actual burden of HR+ and HER2− mBC is scarce. Recently, it has been estimated that 44,642 women were living with mBC in 2019 in Brazil. Of these, 61% were initially diagnosed with stage I-III disease and later progressed to mBC. The remaining patients were diagnosed initially with de novo stage IV disease.^61^ As expected, the majority (58%) of cases are HR+ and HER2−, whereas 25% are HER2+ and 16% are triple-negative BC. Consequently, it is estimated that 25,991 Brazilian women are currently living with HR+ and HER2− mBC, 11,316 with HER2+ mBC, and 7,335 have metastatic triple-negative breast cancer.^61^

With particular importance to this discussion, we should mention the group of patients with metastatic HER2+ disease. Outcomes for this group have improved significantly with the introduction of new treatment alternatives over the last few years. Facing a similar situation of providing these new drugs to the underserved SUS patients with BC, after an intense and collaborative effort with active participation of many stakeholders, anti-HER2 therapy has been only recently made available for patients in the public system in Brazil.^62^

### Advances in Metastatic HR+ and HER2− BC

Endocrine therapy (ET) is the first-line treatment of choice for patients with HR+ and HER2− mBC with the exception of patients with visceral crisis, which should be treated with chemotherapy.^63^ New strategies for HR+ disease have led to significant improvements in the outcomes of patients with first-line and second-line treatment for mBC. Modulation of endocrine signaling combining ET with CDK4/6i, mammalian target of rapamycin, or PI3K-CA has been shown to be effective in clinical practice and has become the preferred option in different settings.^64^ The first-line use of the combination of CDK4/6i with aromatase inhibitors or fulvestrant evaluated in phase III randomized trials demonstrated significant progression-free survival gains when compared with standard single-agent ET.^65-70^ In premenopausal women treated in the first-line setting, remarkably, a clinically meaningful and statistically significant OS benefit was observed with ribociclib.^71^ Additionally, in second-line ET, although the combinations of CDK4/6i with fulvestrant also resulted in improved progression-free survival, the combinations with ribociclib or abemaciclib resulted in improved outcomes in OS as compared with fulvestrant alone.^69-74^

CDK4/6i has been approved and is available in Brazil as of March 2018.^75^ The introduction and availability of these agents across all levels of the health care system represent a challenge, particularly when considering the survival impact of the new therapies. Arguably, the timely availability to appropriate medications should be considered a priority.

### Guidelines and Regulatory Processes

Although these treatments are transforming the field of BC, yielding more long-term disease control than ever before and prolonging OS, health care systems worldwide are struggling to deliver the benefits while balancing sustainability.^76^ Importantly, we need to consider that drugs represent only part of the whole spectrum of management of patients with mBC and broader health economics assessments are an essential aspect to be considered at the same time.

National Comprehensive Cancer Network guidelines have established a framework for resource stratification and indicate essential services based on three levels of basic, core, and enhanced resources.^63^ For example, although tamoxifen is the treatment recommended as part of the most basic framework for patients with mBC, CDK4/6i in combination with ET may be considered as a treatment option only in the third tier of recommendations.^63^ Similarly, European Society for Medical Oncology and ASCO guidelines consider costs and recommend the use of objective scales to evaluate the real magnitude of benefit provided by a new treatment to prioritize funding, particularly in countries with limited resources.^77^ Although not perfect, these guidelines advance the discussion toward a better decision-making process to ensure that resources are allocated appropriately in resource-limited settings.

The English National Health Service and the National Institute for Health and Care Excellence provide the most comprehensive evaluation for the incorporation of new classes of drugs for HR+ and HER2− mBC. They work in partnership with pharmaceutical companies to address uncertainty about the effectiveness of new cancer treatments. This usually involves the collection of additional data, during a managed access period when patients have the opportunity to receive the treatment.^78^ Additionally, some guidelines indicate the usefulness of price negotiations between pharmaceutical companies and governments and may be good examples of alternatives for nationalized health systems.^79^

The Brazilian Health Surveillance Agency (Agência Nacional de Vigilância Sanitária—ANVISA) is primarily responsible for the initial approval of new technologies in Brazil, but additional steps are required to ensure access. The public health system requires a second approval by the National Committee for Health Technology Incorporation (CONITEC), a health technology assessment commission that serves an advisory role to the Ministry of Health. However, this approval does not guarantee access, particularly in the public health system where fixed reimbursement fees represent an added barrier for the incorporation of new technologies. There have been a number of exceptions where centralized negotiations by the MH have taken place facilitating access to particular medications (ie, thalidomide, imatinib, rituximab, and trastuzumab). For the privately covered population, a second approval by another agency, National Health Agency (ANS), is required for oncological oral drugs only, as intravenous medications do not need this second approval. ANS definitive approval is required for coverage to these oral agents to become mandatory by private health plans. So, although CDK4/6i has obtained regulatory approval by ANVISA and is available for some cases in the SH, definitive ANS approval for these agents for SH patients is still pending. On the other hand, CONITEC has not published any recommendations and these agents are not available at all in the public health system.

### Cost-Effectiveness and Thresholds for Decision Making

In the Brazilian public health system, reimbursement is particularly relevant for cancer treatment. For example, based on international guidelines, a patient with HR+ and HER2− mBC should receive a CDK4/6i in combination with aromatase inhibitor as first-line treatment; however, the reimbursement is set at an extremely low fraction of the cost and patients are treated with what the system deems affordable.

Cost-effectiveness studies provide a guide to the adoption of new treatments in various countries, and health technology evaluations have proposed thresholds for the incorporation of these innovations.^80^ A comparison between various countries and their threshold is shown in Table 2.^81-84^ Although there is no established threshold for decision making in Brazil, either for public or private health care, past uptake of new technologies occurred at boundaries between one and three GDP per capita/disability-adjusted life-years.^85,86^

**TABLE 2:**
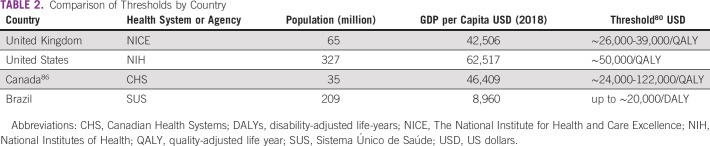
Comparison of Thresholds by Country

Assuming the current costs of the new drugs in Brazil, the costs of treating the estimated 26,000 women with HR+ and HER2− mBC with CDK4/6i would be substantial.^61^ In 2018, the public health system's total budget for systemic therapy for the whole population with all types of cancer was about $658 million US dollars. With the current budgetary situation, it is impossible to continually include innovations within the public health system. Therefore, structural changes are imperative to facilitate patient access to these new and important therapies. Probably, this is a similar challenge for other countries as well.

### Selected Models and Strategies for Financing New Technologies in the Public System

Financing new technologies in the public system is a particular issue in LMICs, and funding of medicines for patients with cancer is a unique challenge because of their usually high costs.^87^ Pricing and reimbursement may have different approaches. Some countries first assess the level of innovation before negotiating prices, whereas others base reimbursement and funding decisions on economic criteria such as cost/quality-adjusted life-year.^88^ Clearly, national pricing strategies should be strengthened to face the incorporations of new technologies.^89^

Several initiatives have addressed cost reduction, prices, and the financial pressure in the health system. Financial schemes such as discounts, rebates, and price volume agreements are among the most common; these are easy to administer and are usually confidential. But this leads to difficulties when comparing prices among countries or care units, resulting in added difficulties in negotiations. Transparency of financial schemes and negotiation processes could significantly affect technology incorporation and use.^89^

In parallel, a number of models have been developed addressing different aspects of the drug-incorporation process. Recently, performance-based schemes have been introduced in some countries, including outcomes as a measure of performance conditioning the reimbursement. These are complex systems requiring the generation of a huge amount of data and a specific and complex information technology infrastructure to be able to follow and evaluate outcomes in a uniform and consistent manner. A further challenge is the potential interference of bias in outcomes analysis, a potentially common situation in cancer treatment. Developing countries usually lack these established information technology capabilities. Furthermore, as an added issue, regulatory laws about personal data privacy may interfere in this process. Difficulties in collecting and analyzing the data and privacy and paucity of clinically relevant outcomes—instead of surrogated end points—have decreased the enthusiasm for performance-based negotiations.^90^

Real-world data may also support decisions about new drugs. Exploring information generated by analysis of data and outcomes collected in the real-word scenario may end up complementing or even replacing outcomes measures, with potentially less bias, and contribute to price reduction negotiations.^91,92^

Managed entry agreements are agreements between the manufacturer (firms that sell health technologies or drugs) and the health care payer that are introduced when prices and/or reimbursement are not decided on by the two parties because of uncertainties about the clinical evidence and/or cost-effectiveness of the product. This method is often applied to facilitate the adoption of technologies by sharing the cost of uncertainty between the payer and the seller, an attractive mechanism for improving access to high-cost and innovative drugs. Managed entry agreements allow a firm to sell a technology while addressing the uncertainty of the performance or budget impact and are frequently also referred to as risk-sharing agreements. These agreements are used in at least two thirds of Organisation for Economic Co-operation and Development countries and European Union members.^93^

Private public partnerships are defined by the WHO as “any informal or formal arrangement between one or more public sector entities and one or more private sector entities created in order to achieve a public health objective or to produce a health-related product or service for the public good.” In this type of agreement, the partners share risks and, in the process, may exchange intellectual property and financial or human resources.^94^

Pooled procurement is another mechanism that has been used to attempt to reduce unit purchase prices. It allows several buyers, either institutions in a single country or health agencies across countries, to collectively negotiate lower prices from developers by combining their bulk purchasing power into a larger purchase commitment on behalf of the pool of buyers.^95^

Although it is clear that there is not one solution that can address all the complexities of technology incorporation in an individual country, these alternatives should be seen as positive attempts to frame the discussion and the generation of much needed context-centered proposals.

### Potential Solutions to Increase Access to Innovative Treatment for Patients With Cancer in Brazil

Affordability and sustainability of new technologies and innovations are currently at the center of any public health system debate. Definition of value, cost-effectiveness and benefit analysis, thresholds, and other measures to support equitable and ethical decisions are continuously evolving. The meaningful clinical outcomes seen with new treatment combinations for patients with HR+ and HER2− mBC are an example of these challenges, creating the need to bring this specific issue to the forefront of the discussion in Brazil.

Decision making for inclusion of new drugs is a complex process and must be addressed based on the political, economic, and social context of each country and requires the participation of key stakeholders. Some countries have implemented various alternatives for new technology incorporation into resource-limited settings, benefiting a significant portion of the population. For instance, some focus on cost-effectiveness alone, whereas others may focus on budget impact alone or both in combination.^96^ In resource-limited countries, budget impact remains the top priority; this prioritization can sway the decision-making process heavily, especially in the context of oncologic therapeutics, which is frequently disproportionally expensive.^97^ Global frameworks may provide different perspective on what impact should be prioritized; however, as previously mentioned, specific solutions are context dependent and should be developed locally.

**FIG 1:**
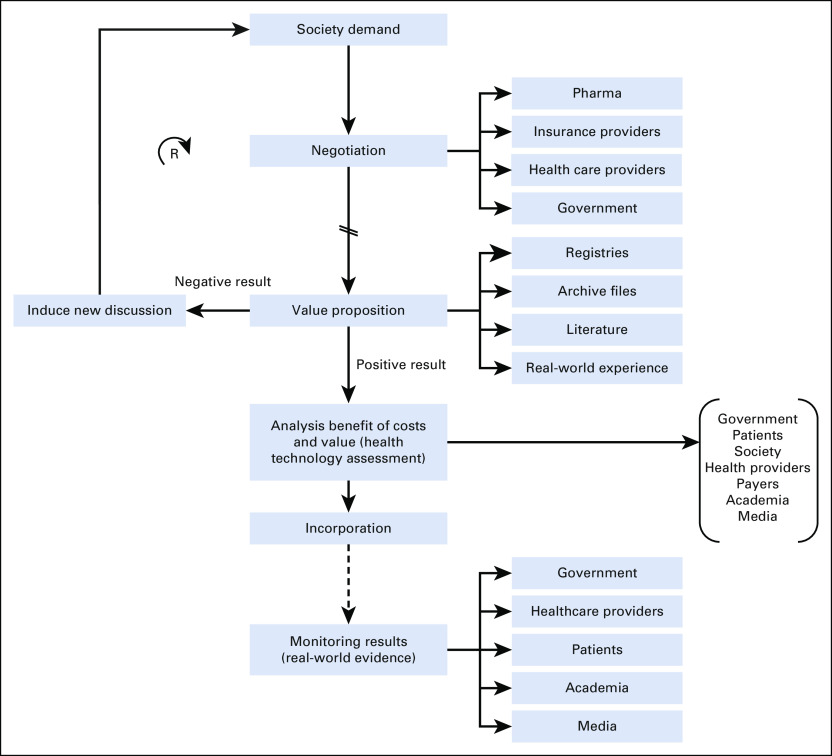
Proposed process and stakeholders for advanced regulatory approval and drug reimbursement in Brazil. “R” on the left side of the figure indicates that this may be a revolving process. Black arrows indicate that the process moves as quickly as processes allow. Two slashes through the black arrow indicates that the process is delayed. Dotted arrow indicates that the process takes place over time.

Recognizing that we are addressing an issue with broader implications in the healthcare system, the panel recommends implementing a pilot program that could potentially be applied to other similar situations, promoting access to ongoing innovative treatment alternatives. The suggested program seeks to overcome previously mentioned Brazilian-specific obstacles. This framework, outlined in Figure 1, can be critical for ensuring access to novel therapies for mBC and, subsequently, other diseases in the country. For this pilot to be successful, key stakeholders fundamental to participate in the initial discussion should include government, pharmaceutical companies, medical societies, patients, payors, health care providers, media, and academia. This group should be convened by the government (Ministry of Health) to engage in the development of strategies to address specific obstacles, generate solutions for the treatment of this specific patient population, and define the timeline for implementation.

This roadmap includes proposed areas and steps that should be followed toward a successful implementation of this pilot (Fig 1).

### Roadmap

#### Society demand (defining the intervention and target population)

Defining the societal demand for an intervention is essential for policymakers and for all stakeholders participating in the process. We should clearly describe the target disease burden that is being considered and indicate the need for and the potential benefits of an intervention. Disease burden is the impact of a health problem on a given population and can be measured by several indicators including mortality, morbidity, and cost (financial and societal). It is often measured using two widely accepted indicators that consider both death and morbidity and facilitate comparison. These are quality-adjusted life-years and disability-adjusted life-years. Measuring disease burden is important to prioritize health action, plan for preventive action, and assess performance of health care systems.^98^ Cancer registry data and data from population-based registries can provide necessary information to quantify the problem.^99^

#### Negotiation

The initial negotiations must include the pharmaceutical industry, insurance providers, health care providers, and government and should generate a comprehensive discussion incorporating all stakeholder's perspectives. Negotiations should be based on the market size to provide a baseline treatment cost that can be used to complete health technology assessments. During negotiations, innovative solutions such as managed entry agreements, risk-sharing schemes, private-public partnerships, technology transfer opportunities, and pooled procurement should be explored. As these potential solutions become more popular across nations, the demand will grow for a platform that can accommodate all stakeholder needs fairly.

#### Value proposition and health technology assessment

*Value propositions* can be generated based on the cost and clinical benefit of a certain product and depend largely and initially on the treatment options available for the disease. Currently, value propositions should be addressed on a community level with evidence showing how a product will affect the system and its management.^100,101^ An example of a value proposition is that, for the same cost and the same cancer, a given treatment demonstrated a 10% increase in patients achieving 2-year progression-free survival compared with the standard of care or the results of a competitor. *Health technology assessment* refers to a multidisciplinary process where a systematic evaluation of properties, effects, and/or impacts of health technologies and interventions is performed. It covers both the direct, intended consequences of the technology and the indirect, unintended consequences.^102^ This process is used to determine the value of a health technology and informs policy decision making in health care to promote an equitable, efficient, and high-quality health system.^102^

In this step, a value proposition is generated through objective data found in registries, archive files, literature, and real-world experience. This initial proposal should highlight the potential benefits and opportunities of the technology being analyzed. From this value proposition, government agencies (CONITEC in the case of Brazil) should perform a formal analysis of costs and value. This process should be based on predefined and transparent procedures analyzing efficacy, accuracy, effectiveness, safety, cost-effectiveness, and budgetary impact of the specified technology.^103^ This step is critical, as it informs all stakeholders on the potential impact of the technology's implementation for public patients (SUS in Brazil), health care professionals, and industry. Several articles have dissected the workflow of CONITEC and how it could be improved; however, that is beyond the scope of this article.^103^

#### Incorporation

If the health technology assessment results in a positive outcome, incorporation should be implemented. At this point, difficulties surrounding reimbursement, pricing, safety, indications, and others should be identified and addressed. Importantly, practice guidelines for rational use of the new technology must be simultaneously developed and disseminated to guide all relevant stakeholders. Ideally, the Ministry of Health should have clearly defined procedures, streamlined with the health technology assessment outcomes for synergy and clarity. A clearly defined process with multidisciplinary participation should help and support the implementation of the new technology. Throughout incorporation, logistical and practical issues related to treatment delivery and patient adherence must be addressed.

#### Measure or monitor outcomes and share results

Monitoring health outcomes and impacts is vital to achieving value-based health to ensure higher value for patients and sustainability of the health care system. This includes result-based financial indicators that monitor a change in the health status of a group or population attributable to an intervention and includes the measures of morbidity and mortality. The use of clinical registries is crucial for this end, and these are widely used to evaluate outcomes.^104^ Specifically, much needed comprehensive national cancer registries could provide key data to monitor the impact of the proposed interventions and achieve quality improvement and public accountability. As target populations are closely monitored, key stakeholder must be informed on a regular basis on the results of these analyses.

The development of new technologies, changes in population demographics, and variations in the market landscape, among others may drive a review of the pilot program requiring adaptation for the next implementation round.

In conclusion, this panel has addressed particular issues related to the lack of access to cutting edge therapies in the public health system in Brazil. Availability of innovations in cancer is of global concern, and thus, the specific issues discussed are not exclusive to this country. With increasing health care costs and limited resources, there is a clear need to discuss innovative strategies to allow for the benefits of newly developed technologies to reach the population they were developed for. The proposed framework attempts to comprehensively address access to innovation in cancer care; however, it is not intended as a one-size-fits-all solution and can and should be tailored on a country-by-country basis.
